# DEGRO practical guideline for central nervous system radiation necrosis part 1: classification and a multistep approach for diagnosis

**DOI:** 10.1007/s00066-022-01994-3

**Published:** 2022-08-29

**Authors:** Denise Bernhardt, Laila König, Anca Grosu, Benedikt Wiestler, Stefan Rieken, Wolfgang Wick, Jens Gempt, Sandro M. Krieg, Friederike Schmidt-Graf, Felix Sahm, Bernhard Meyer, Bernd J. Krause, Cordula Petersen, Rainer Fietkau, Michael Thomas, Frank Giordano, Andrea Wittig-Sauerwein, Jürgen Debus, Ghazaleh Tabatabai, Peter Hau, Joachim Steinbach, Stephanie E. Combs, Expert Panel of the German Society of Radiation Oncology (DEGRO)

**Affiliations:** 1 Klinik und Poliklinik für Radioonkologie und Strahlentherapie, Klinikum rechts der Isar, Technische Universität München, Ismaninger Str. 22, 81675 München, Germany; 2 Institute of Radiation Medicine (IRM), Department of Radiation Sciences (DRS), Helmholtz Zentrum München (HMGU), Ingolstädter Landstr. 1, 85764 Oberschleißheim, Germany; 3 Partner Sites Munich, Freiburg and Heidelberg, Deutsches Konsortium für Translationale Krebsforschung (DKTK), Heidelberg, Germany; 4 Klinik für Radioonkologie und Strahlentherapie, Universitätsklinikum Heidelberg, Im Neuenheimer Feld 400, 69120 Heidelberg, Germany; 5 Heidelberger Ionenstrahltherapie-Zentrum (HIT), Im Neuenheimer Feld 450, 69120 Heidelberg, Germany; 6 Heidelberg Institute of Radiation Oncology (HIRO), Im Neuenheimer Feld 400, 69120 Heidelberg, Germany; 7 Clinical Cooperation Unit Radiation Oncology, German Cancer Research Center (DKFZ), Im Neuenheimer Feld 280, 69120 Heidelberg, Germany; 8 National Center for Tumor diseases (NCT), Heidelberg, Germany; 9 Department of Radiation Oncology, Medical Center—University of Freiburg, Faculty of Medicine, University of Freiburg, Robert-Koch-Str. 3, 79106 Freiburg, Germany; 10 Department of Neuroradiology, School of Medicine, Klinikum rechts der Isar, Technical University Munich, Munich, Germany; 11 Clinic of Radiotherapy and Radiation Oncology, University Medical Center Göttingen, Robert-Koch-Str. 40, 37075 Göttingen, Germany; 12 Clinical Cooperation Unit Neurooncology, German Consortium for Translational Cancer Research (DKTK), German Cancer Research Center (DKFZ), Heidelberg, Germany; 13 Department of Neurology and Neurooncology Program, National Center for Tumor Diseases, Heidelberg University Hospital, Heidelberg, Germany; 14 Department of Neurosurgery, School of Medicine, Klinikum rechts der Isar, Technical University Munich, Munich, Germany; 15 Department of Neurology, School of Medicine, Klinikum rechts der Isar, Technical University Munich, Munich, Germany; 16 Department of Neuropathology, University Hospital Heidelberg and CCU Neuropathology, German Consortium for Translational Cancer Research (DKTK), German Cancer Research Center (DKFZ), Heidelberg, Germany; 17 Department of Nuclear Medicine, Rostock University Medical Centre, Rostock, Germany; 18 Department of Radiotherapy and Radiation Oncology, University Medical Center Hamburg-Eppendorf, Martinistr. 52, 20246 Hamburg, Germany; 19 Department of Radiation Oncology, University Hospital Erlangen, Friedrich-Alexander-Universität Erlangen-Nürnberg, Erlangen, Germany; 20 Comprehensive Cancer Center Erlangen-European Metropolitan Region of Nuremberg (CCC ER-EMN), Erlangen, Germany; 21 Department of Thoracic Oncology, Thoraxklinik at Heidelberg University Hospital, Heidelberg, Germany; 22 Department of Radiation Oncology, University Hospital Bonn, Bonn, Germany; 23 Department of Radiotherapy and Radiation Oncology, University Hospital Jena, Bachstr. 18, 07743 Jena, Germany; 24 Department of Neurosurgery, University Hospital Tuebingen, Eberhard Karls University Tuebingen, Tuebingen, Germany; 25 Center for Neuro-Oncology, Comprehensive Cancer Center Tuebingen Stuttgart, University Hospital Tuebingen, Eberhard Karls University of Tuebingen, Tuebingen, Germany; 26 Department of Neurology, Eberhard Karls University of Tuebingen, Tuebingen, Germany; 27 Department Interdisciplinary Neuro-Oncology, Eberhard Karls University of Tuebingen, Tuebingen, Germany; 28 Wilhelm Sander-NeuroOncology Unit and Department of Neurology, University Hospital Regensburg, 93053 Regensburg, Germany; 29 Dr Senckenberg Institute of Neurooncology, University Hospital, Frankfurt am Main, Germany; 30 Member of the German Center for Lung Research (DZL), Translational Lung Research Center Heidelberg (TLRC-H), Heidelberg, Germany

**Keywords:** Radioation necrosis, Reirradiation, Bevacizumab, Brain metastases, Glioma

## Abstract

**Purpose:**

The Working Group for Neuro-Oncology of the German Society for Radiation Oncology in cooperation with members of the Neuro-Oncology Working Group of the German Cancer Society aimed to define a practical guideline for the diagnosis and treatment of radiation-induced necrosis (RN) of the central nervous system (CNS).

**Methods:**

Panel members of the DEGRO working group invited experts, participated in a series of conferences, supplemented their clinical experience, performed a literature review, and formulated recommendations for medical treatment of RN including bevacizumab in clinical routine.

**Conclusion:**

Diagnosis and treatment of RN requires multidisciplinary structures of care and defined processes. Diagnosis has to be made on an interdisciplinary level with the joint knowledge of a neuroradiologist, radiation oncologist, neurosurgeon, neuropathologist, and neuro-oncologist. A multistep approach as an opportunity to review as many characteristics as possible to improve diagnostic confidence is recommended. Additional information about radiotherapy (RT) techniques is crucial for the diagnosis of RN. Misdiagnosis of untreated and progressive RN can lead to severe neurological deficits. In this practice guideline, we propose a detailed nomenclature of treatment-related changes and a multistep approach for their diagnosis.

## Introduction

Improvements in systemic therapies, especially targeted therapies, and radiotherapy (RT) techniques have led to prolonged survival in cancer patients over the past decade. Oligometastatic patients receive more radical local treatments and stereotactic radiotherapy (SRS) is frequently used in this setting. Furthermore, longer survival leads to an increased absolute risk for cancer patients to develop brain metastases (BM) during the course of their disease. As whole-brain radiotherapy (WBRT) is associated with significant neurocognitive decline compared to SRS, SRS has been explored and increasingly utilized for selected patients with multiple BMs [1–3]. But also patients with primary brain tumors (e.g., glioma, glioblastoma, etc.) receive more reirradiation and molecular analyses allow for targeted therapies in selected patients, sometimes in combination with RT [4–7]. As a result, long-term side effects have become more prevalent—typically identified on posttreatment imaging including computed tomography (CT) and magnetic resonance imaging (MRI) as contrast-enhancing lesions (CEL). Contrast enhancement can represent a variety of pathophysiologies including treatment-associated effects such as radiation necrosis (RN) or blood–brain barrier disruptions (BBD); sometimes also referred to as pseudoprogression or late radiation tissue injury (LRTI) [8, 9]. Unfortunately, differentiation between RN, BBD, and tumor progression is extremely challenging, and the term itself is often misleading due to the continuous temporal overlap. The aforementioned more radical radio-oncological approaches in glioma and other cancer therapies involving the central nervous system (CNS) in combination with prolonged survival lead to an increase in the incidence of CEL. The clinical course may vary and while some of these CEL are clinically subtle and demonstrate long-term radiological stability, others may display a more acute and “malignant” course, leading to substantial symptoms and therefore requiring prompt action, while also challenging physicians to differentiate between treatment-associated effects and true tumor progression. In addition to the variations in clinical behavior, the nomenclature is not used consistently in the literature. The terms “radiation necrosis,” “pseudoprogression,” and “blood–brain barrier disruptions” are used synonymously both in tumor boards and throughout the literature, despite describing different clinical entities [10].

Currently there is no defined guideline for the treatment and diagnosis of RN. Several guidelines already recommend the use of steroids and bevacizumab in the treatment of RN, although there are no defined treatment algorithms [11, 12]. This lack of consensus was identified by the German Society for Radiation Oncology (DEGRO) and the DEGRO board therefore mandated the DEGRO working group to establish a practice guideline. In 2020, a position paper about the use of bevacizumab and the treatment of RN was already established and published by the DEGRO society [13]. The aim of this practice guideline is to propose a distinct nomenclature and develop a practical approach for the diagnosis and treatment of new radiation-induced CEL (RN vs. BBD). In this practice guideline, we have integrated the limited results from contemporary clinical trials and the available retrospective data. The implementation of this guideline requires multidisciplinary structures of care and defined processes of diagnosis and treatment of CEL.

## Methods

This guideline was prepared by an expert panel of the German Society of Radiation Oncology (*Deutsche Gesellschaft für Radioonkologie*, DEGRO) Working Group for Neuro-Oncology (AG NRO) in cooperation with members of the Neuro-Oncology Working Group of the German Cancer Society (DKG-NOA). The guidelines subcommittee recruited a panel of recognized experts from the field of neurosurgery, neuroradiology, and neuro-oncology/neurology. This task force represents all disciplines involved in the diagnosis and care of patients with CNS RN/BBD. We retrieved references published in English on PubMed with the search terms “radiation necrosis” alone and in combination with “avastin,” “bevacizumab,” “steroids,” “radiosurgery,” “stereotactic,” “re-irradiation,” “vascular endothelial growth factor (VEGF),” “immunotherapy,” or “dexamethasone,” from January 1, 2000, to November 1, 2021. We also identified publications through searches of the authors’ own files. Screening and initial eligibility were addressed by two authors (DB, LK), consulting others for disagreement resolution. Panel members of the DEGRO and experts participated in a series of virtual conferences and circular emails and supplemented their clinical experience and formulated recommendations for the treatment and diagnosis of RN in clinical routine. The treatment recommendations were formed by full consensus of the participating experts.

## Results

### Incidence and pathophysiology of RN and BBD

The true incidence of RN and BBD is hard to estimate and varies according to the diagnostic criteria and modality used as well as treatment-associated factors and lies between 5–30% [8–10]. Moreover, the risk of development is influenced by the time, type, and temporality of systemic treatments; applied (cumulative) radiotherapy dose; tumor volume; and type of cancer. Statistics must be taken with a grain of salt since much of the published data are reliant on radiological rather than pathological endpoints.

**Fig. 1:**
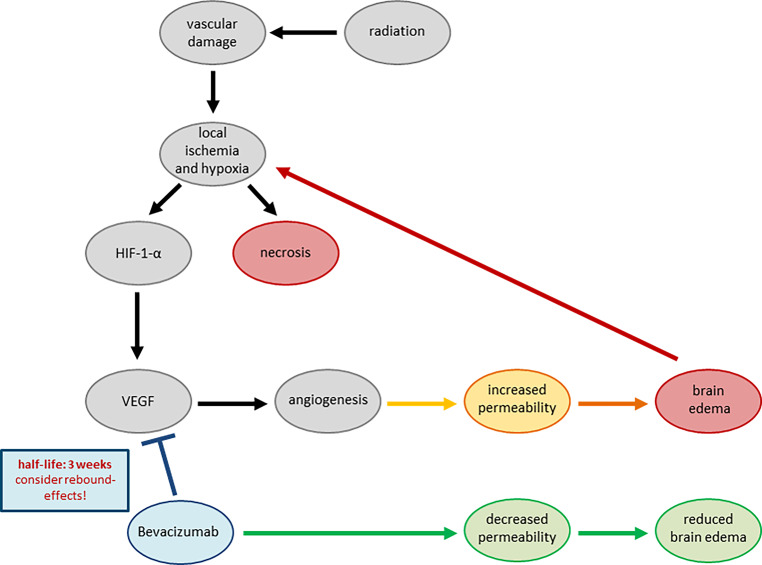
Pathogenesis of blood–brain barrier disruptions and radionecrosis with the targeting point of bevacizumab as an inhibitor of vascular endothelial growth factor, therefore being a potent effector for disrupting the vicious cycle (adaption of Fig. 1 from Zhuang et al. [15])

Three distinct types of radiation injury can be recognized: acute (during or shortly after radiation), subacute or early-delayed (typically up to 12 weeks after radiation), and late (months to years after completion of radiation) [14]. Two theories behind the pathophysiology of RN and BBD have emerged over the past few years, although a multifactorial cause seems most likely [15, 16]. The acute injury during or immediately after completion of RT is mostly reversible and secondary to edema associated with increased capillary leakage. BBD likely results from transiently increased permeability of the tumor vasculature and inflammation induced by previous therapies [17]. If tissue is submitted to histological analysis, paravascular edema and inflammatory infiltrates might be observed. However, the assessment can be hampered by similar alterations subsequent to the proceeding surgery. Since radiation can induce cellular atypia presenting similar to neoplastic cells, molecular analysis for pathognomonic tumor alterations may be required when morphology is not indicative. Nevertheless, both mechanisms are closely interlinked and mutually dependent, which can be observed at the cellular level: vascular tissue damage due to RT leads to local ischemia and hypoxia and increased secretion of HIF-1-alpha, which leads to increased liberation of vascular endothelial growth factor (VEGF) [14, 15, 18]. VEGF is the main driver for angiogenesis of abnormal vessels with increased permeability, which promotes exudation and thus brain edema. This in turn causes additional ischemia and hypoxia, leading to a vicious positive feedback loop and ultimately ending in brain necrosis. Extensive research has explored cellular mechanisms that could be targeted in an effort to manage this clinical syndrome [19]: Gonzales et al. first reported the treatment efficacy of bevacizumab [20], a monoclonal antibody binding VEGF and therefore a potent target to disrupt this vicious cycle. A delayed (late) injury follows months after RT, is irreversible in most cases, and is caused by direct injury to glial cells. Histological analysis may reveal reduced cellularity of neurons and glia or vacuolization, but also gliosis, hyalinized vessels, or fibrinoid necrosis of endothelium. Wider RN with involvement of the parenchyma is challenging to diagnose since necrosis occurs in most high-grade CNS tumors even without therapy. In contrast to tumor necrosis, RN is often coagulative, may include areas of dystrophic calcification, and remnants of hyalinized and necrotic vessels may be observed. RN can be surrounded by hypocellular tissue, whereas tumor necrosis is mostly adjacent to highly cellular and proliferating neoplastic cells. (Fig. 1).

With conventional fractionation (1.8–2 Gy per fraction) and doses ≤ 60 Gy, symptoms of acute radiation injury are typically mild and self-limiting and mostly completely resolve without therapeutic intervention [18]. BBD frequently presents with no or few symptoms, is often self-limiting and slowly changing in size, whereas RN on the other hand often presents with notable symptoms, a consecutive need of therapeutic intervention, and rather fast progression/volumetric change over time. Patients may suffer from symptoms due to increased intracranial pressure (headache, nausea, and dizziness), seizures, loss of cranial nerve function, or other symptoms specific to the neuroanatomical location of the lesion. Since symptoms in patients with RN are often caused by edema, they often respond well to corticosteroid therapy, which might be a possible factor for differentiating between RN and tumor progression.

### Nomenclature—differentiation between CELs

Given the variety of terms used to differentiate clinical and radiological CEL, here we propose objective criteria to define BBD versus RN (Table 1):

1. *Blood–brain barrier disruptions (BBD; also referred to as “pseudoprogression”) and nonmeasurable, speckled contrast-enhancing lesions (SCEs).*
  Radiation-induced BBD occurs as CEL in primary and secondary brain tumors, both in field and out of field relative to the high-dose radiation volume. The maximal tolerated dose with a 5% rate within 5 years (TD5/5) for brain necrosis (= 60 Gy EQD2) is usually not exceeded (according to Emami et al. [21] and QUANTEC [22, 23]). BBD or pseudoprogression occurs predominantly within the first 6 months after (chemo)radiotherapy.
  Small and clinically asymptomatic SCEs can occur in glioma patients during the course of their disease without immediate relation to prior therapy [24]. SCEs occur more frequently in World Health Organization grade 2 and 3 astrocytoma and oligodendroglial tumors and gliomas with an isocitrate dehydrogenase (IDH) mutation. In patients with glioblastoma, SCEs were associated with a favorable prognosis, which was also observed in the subgroup of patients with glioblastoma with IDH wildtype status [24, 25]. These SCEs in glioma patients typically develop temporally distant from RT (6–18 months), may vary in their location over the follow-up period, and are typically temporary and often asymptomatic. At the cellular level, these changes are caused by small capillary leaks with or without edema [24]. SCEs mostly remain stable or dissolve with no further treatment. The manifestation of a spontaneous immune reaction rather than a new tumor manifestation is discussed as potential causes of SCEs.
2. *Radiation necrosis (RN) occurs in the high-dose-treated region. The CEL are caused by significant capillary leakiness but also direct glial damage and therefore often present with a large zone of edema leading to pronounced clinical symptoms. If not treated promptly, these changes are irreversible and may even be lethal.*
  In the literature, typical RN is described to occur later in the course, usually 6–18 months after (chemo)radiotherapy [14, 26].
  Early RN: it is important to take radiation technique, total dose, and fractionation into account. If the TD5/5 has been exceeded by large numbers (e.g., SRS, Re-RT) and the CEL occurs directly or shortly after RT (< 6 months), potentially showing a rapid progression, early RN can be more likely than BBD/pseudoprogression [27, 28].
  Late- or ultra-late RN: with prolonged survival of cancer patients in the past decade (e.g., ALK-mutated NSCLC), the incidence of (ultra-) late RN has increased, and it is important to notice that RN can occur several years and even decades after radiotherapy, especially after high-dose SRS. Late-RN is often misinterpreted as tumor progression, which can lead to discontinuation of a successful treatment, unnecessary operations, or re-irradiation and unnecessary systemic therapies, and can therefore be extremely harmful for a patient. Misdiagnosis of untreated or mistreated progressive RN can lead to severe neurological deficits due to further progression of the RN [29–31].
3. *Late tissue effects. *Additionally to BBD and RN, radiation-induced late effects include white matter changes [32, 33], cerebral atrophy, and vascular lesions such as lacunar infarcts and parenchymal calcifications [34]. The interval between therapy and time to occurrence varies from months to several years. CNS injury, as demonstrated by subclinical white matter changes, is relatively common and can even manifest after chemotherapy alone.
4. *Mixed forms. *Since both entities are closely linked with each other at the pathogenesis level, BBD may show a fluent transition to RN and sometimes a clear distinction between RN and BBD is not possible or reasonable (see Table 1). In gliomas, especially recurrent glioblastoma, a mixed form of BBD, RN, and progressive disease is common.

**Table 1:** Nomenclature with characteristics for differentiating between BBD and RN

	BBD	BBD	BBD along isodoses	Early RN	Typical RN	(Ultra-) late RN
RT	Primary RT, normofractionated	High-dose primary RT or Re-RT	Single-fraction SRS or hypofractionated SRS, CyberKnife, Gamma Knife	After exceeding the TD5/5 by a large number (Re-RT with photons, SRS or C12)	Possible after all forms of RT	Possible after all forms of RT
Dose range	≤ 60 Gy (54–60 Gy)	> 60 Gy cumulative or high fraction dose	Ablative doses (e.g., 20/18 Gy single dose, Cavity SRS)	Cumulative doses EQD2 > 100 Gy TD5/5 is exceeded widely	Clear dose–volume dependency, TD5/5 can be exceeded	Clear dose–volume dependency, TD5/5 can be exceeded
Time after RT	Typically 1–6 months after RT (“pseudogrogression”) Can occur later (6–18 months)	Typically 1–6 months after RT (“pseudoprogression”) Can occur later (6–18 months)	Typically 3–6 months after RT (“pseudoprogression”) Can occur later (6–18 months)	Early, often 1–6 months after RT	6–18 months	> 18 months–several years
Special considerations	CEL in association with ventricular system after protons (distal end of beams, increase of RBE) Frontal or temporal lobe (protons: lateral beam application)	CEL in association with ventricular system after protons (distal end of beams, increase of RBE) Frontal or temporal lobe (protons: lateral beam application)	CEL according to isodoses, clear dose–volume dependency, central necrosis of tumor tissue is the desired treatment effect in tumors, especially brain metastasis. Association with ventricular system possible. Higher risk in patients treated with immunotherapy concomitantly	Often large edema, central necrosis	Usually mixed form of BBD and RN	Often misdiagnosed as progression, often associated with immunotherapy
Progression pattern	Slow, fluctuating, usually self-limiting, reversible Progression into RN is rare	Slow, fluctuating, often reversible Progression into RN is possible	Fluctuating, middle, often self-limiting and reversible, Progression into RN is possible	Rapidly, can be tumor like, Irreversible	Often progressive, can be tumor like, Irreversible	All forms of progression, irreversible
Symptoms	Typically no/few symptoms, small–medium edema possible	Small–medium edema possible, symptoms usually not severe	Small–medium edema possible, symptoms usually not severe	Small–large edema possible, often associated with large edema, symptoms can be severe	Small–large edema possible, symptoms can range from asymptomatic to severe	Small–large edema possible, symptoms can range from asymptomatic to severe

*RT* radiotherapy, *CEL* contrast-enhancing lesions, *RBE* relative biologic effectiveness, *BBD* blood–brain barrier disruptions; Gamma Knife, ELEKTA, Sweden; CyberKnife, Accuray Incorporated, Sunnyvale, CA, USA

### Diagnostic multistep approach

#### General recommendations

Biopsy is still regarded as the diagnostic gold standard to differentiate between tumor progression (TP) and RN/BBD, but cannot be performed in all patients due to the location of the lesion or the general performance status of the patient. Further, the diagnosis is difficult despite histopathologic analysis as a recent study revealed, since a biopsy might be not representative enough or yield insufficient amounts of tissue. Only marginal reproducibility was found when pathologists were asked if they were able to provide a final diagnosis (BBD vs. RN vs. progression) in patients with suspected recurrent glioblastoma, also because necrosis itself is a characteristic for glioblastoma [35]. Definitive diagnosis without pathology is difficult, and until the present day, no diagnostic modality provides absolute certainty. With respect to the following points, it is important to mention that the diagnosis has to be made on an interdisciplinary level with the joint knowledge of a neuroradiologist, neuropathologist, radiation oncologist, neurosurgeon, and neuro-oncologist, and revision of the applied treatments (radiation plan, immunotherapies, chemotherapies, etc.). This is one of the reasons why we recommend the multistep approach as an opportunity to review as many characteristics as possible to improve diagnostic confidence. Because many of the new CEL represent a mixture of tumor cells and radiation injury, the goal is to identify the predominant component.

#### Diagnostic imaging

Diagnostic imaging should be done on a regular basis during follow-up (FU) using MRI with and without gadolinium contrast. First signs that may be seen are an increase in the T2-FLAIR signal corresponding to edema, which often occurs before the development of CEL. Contrast-enhanced MRI is the basis of brain imaging, but its specificity for the differentiation between blood–brain barrier disturbances related to either the treatment or to tumor progression is low [36–38]. T1w contrast-enhanced sequences show damage in the blood–brain barrier with contrast leakage to surrounding normal brain tissue, therefore it can be seen in BBD and the marginal zone of RN, as well as in true tumor progression. Compared to BBD, CE in RN often shows rapid growth, very similar to a tumor-like growth pattern. Areas of CE and high T2-FLAIR typically show a decrease in regional cerebral blood volume (rCBV) in perfusion imaging and an increase in the apparent diffusion coefficient (ADC) in diffusion imaging, which can be helpful in distinguishing them from residual tumor/recurrence, which typically present with increased rCBV and decreased ADC as correlates of neoangiogenesis and hypercellularity. Nevertheless, both can be seen in the central location of the tumor and demonstrate the difficulty of diagnosis based on imaging alone [39].

Further advanced complementary diagnostic tools like MR spectroscopy (MRS) or positron-emission tomography (PET) may support clinical decision-making. MRS may show low choline, creatinine, and N‑acetylaspartate (NAA) peaks [40, 41]. A correlation with the initial behavior of the tumor might be beneficial when differentiating from tumor progression (e.g., low-grade glioma with no or low-contrast enhancement).

The Response Assessment in Neuro-Oncology (RANO) working group established guidelines to improve the assessment of tumor response in gliomas, specifically in the context of clinical trials [42, 43]. According to the RANO criteria, in the first months after completion of chemoradiotherapy, tumor progression can only be radiologically defined if there is new enhancement outside the radiation field (beyond the 80% isodose line). If the area of new or increased enhancement occurs inside the radiation field, pseudoprogression (BBD) is more likely and further evidence of tumor progression is required by histopathologic sampling or follow-up imaging showing further progression of contrast-enhancement. Additionally, factors that might influence CEL and therefore BBD and RN are operative procedures and concomitant chemotherapy [44] or immunotherapeutic agents [45, 46]. The RANO working group also published guidelines for the evaluation of response in glioma patients who underwent immunotherapy treatments [42]. If the lesion developed within ≤ 6 months after starting immunotherapy and the patient has no new or progressive neurologic symptoms, follow-up imaging is required for diagnosis confirmation. RANO criteria now include the use of dexamethasone as well as information about the radiotherapy target volume (e.g., 80% isodose line). Additional information about radiotherapy like the biological dose, re-irradiation, and radiotherapy technique are not considered in the RANO criteria but are crucial for the diagnosis of RN. The location of a CEL relative to the irradiated tumor and the radiation field is the most important factor in deciding whether the lesion is a new abnormality secondary to radiation. Amino acid tracers are applied for RT planning [47], but also for the differentiation of recurrent or progressive disease and pseudoprogression or RN after initial RT [48–52], as published by the PET RANO group [53]. Several FET or F‑DOPA PET studies have suggested that a differentiation between BBD or RN and relapse can be obtained with a high diagnostic accuracy between 80–90% [54–57] and dynamic FET PET acquisition may further increase diagnostic value [51, 54, 55, 58, 59].

In analogy to gliomas, brain metastases (BM) can also be visualized by PET imaging [59]. PET imaging has evolved as a complementary imaging tool for the differentiation of true progression from CEL [60–62], and the use of PET in brain metastases was also recently recommended by the PET RANO group [59]. However, PET differentiation is less used in clinical routine in brain metastases compared to glioma and cost coverage can be an issue. Moreover, necrosis of tumor tissue is a wanted effect in ablative SRS of BM. Currently, there is no imaging technology to distinguish between brain tissue necrosis and tumor necrosis, and a multistep approach to obtain diagnostic accuracy is needed. To correctly interpret CEL after SRS of BM or cavities after BM resection, the evidence of local tumor control must be understood depending on size/volume of the lesion and applied dose. Local control rates after SRS are typically in the range of > 90% and, therefore, RN is more likely than tumor progression [29]. Depending on the size of the lesion, volume of the treated area, and fractionation, tumor progression can become more likely [1, 63, 64]. Differences in isodose surface selection for target coverage can lead to differences in local control. In day-to-day clinical routine, isodose curves, target coverage, dose prescription, and detailed information about radiotherapy are often not available to (neuro)radiologists, which is a relevant practical issue for response assessment according to the RANO criteria outside of clinical trials. Neuroradiologists are strongly encouraged to discuss potential radiation-induced changes with radiation oncologists to correctly assess CEL.

Modern image analysis strategies combining PET and MRI have shown promise in the differentiation of tumor progression from BBD or RN. In the future, computational image analysis including automated tumor segmentation and classification might further improve this [65, 66].

#### Correlation of diagnostic images and CEL patterns with RT treatment plans

Correlation of diagnostic images with the radiotherapy treatment plan (offline or online after fusion in the treatment planning system) is one of the main pillars of the diagnostic procedure and should be performed by an experienced radiation oncologist. Regarding RT, several factors should be considered, such as (cumulative) radiation dose, fractionation, prescription (homogeneously vs. inhomogeneously), and treatment technique (IMRT, 3D-CRT, SRS, particle therapy).

*Special considerations of radiation-induced CEL according to RT technique, typical localization, shape, and appearance*:

- BBD after radiotherapy with association to the subventricular zone of the ventricular system can be seen frequently after photon and proton RT with doses that are below the TD5/5 in the marginal area of the RT volume. This may be due to the location of neural stem cells in the ventricular subependymal region [67, 68] that are probably more sensitive to irradiation. In particle therapy, according to the beam arrangement, on the marginal treatment field where an increased RBE (and therefore higher dose with exceedance of the TD5/5) due to the distal edge of Bragg peak is expected, BBD occur more frequently [69–71]. This can often be seen in the temporal lobes and also near the ventricular system. BBD usually occurs within the first 6 months after RT, although BBD associated with the ventricular system can appear later (up to 18 months after treatment), and transition into RN is possible if not diagnosed and treated correctly.
- Typical RN can appear on axial contrast-enhanced T1-weighted MR images as a so-called Swiss cheese or spreading wavefront enhancement pattern [33, 72]. The pattern can be similar to the appearance of a cut pepper, especially in large brain metastases or gliomas with central tumor necrosis.
- Growing CEL often appear after SRS or hypofractionated RT and appearance is clearly dose–volume correlated [1, 32, 73]. These CEL, which start as BBD, can occur quite fast after the end of RT due to the high dose of SRS and may progress rapidly and may easily merge into progressing RN if there is relevant brain tissue damage. Central necrosis of tumor tissue in BM therapy is exactly the effect that is desired in treatment with SRS. To distinguish between the desired necrosis of tumor tissue and an unwanted BBD or RN of the surrounding brain is often not possible, due to the close proximity of tumor and healthy cells.
- RN may appear ubiquitously when exceeding the TD5/5, often in cases of Re-RT with photons, high-single-dose radiosurgery, or particles. They often show a strong CEL with tumor-like and rapid growth patterns with large perifocal edema [21, 22].

*CEL in typical loci after RT of extracranial head and neck malignancies or intracranial extraaxial tumors: patients with extracranial tumors such as chordomas or chondrosarcomas or (recurrent) head and neck cancers (HNC) frequently receive a total cumulative dose that is above the maximum dose constraints for organs-at-risk (OARs) such as the temporal lobe. In these cases, a risk–benefit tradeoff is inevitable due to an unfavorable tumor location near OARs.*

- Advances in radiotherapy technique and the availability of particle therapy enable radiation oncologists to deliver high doses to the target volume. In the (curative) treatment of HNCs like nasopharyngeal cancer, adenoid cystic carcinomas, esthesioneuroblastoma, and chordomas and chondrosarcomas, with close proximity to the healthy brain tissue, radiation-induced frontal and temporal lobe necrosis (TL-RN) is a common complication [26, 74–76]. QUANTEC data reveal a dose–response relationship in the brain where the incidence of RN increases from 3% with a Dmax < 60 Gy to 5% at Dmax = 72 Gy [22]. The total dose needed to gain tumor control usually exceeds 60 Gy and radiation oncologists therefore hazard the consequences of a potential RN. Multiple studies have reported on the dose–volume relationship for temporal lobe necrosis using both photons and protons [73, 77–80]. BBD or RN usually occurs in typical loci at the frontotemporal region or the temporal lobe, depending on the radiation field. The risk for (TL-)RN rises with the use of re-irradiation and in patients with infiltration of the skull base or brain [81]. CEL can easily be misinterpreted as tumor progression, which can lead to harmful consequences for the patients. Therefore, HNC patients with high-dose RT of the skull base or the temporal lobe should be monitored closely by radiation oncologists. Prospective and retrospective data show that treatment with bevacizumab leads to quick symptom relief and radiographic improvement in this setting [26, 82].

*CEL in patients undergoing immunotherapy and targeted therapies:*

- Checkpoint inhibitors (CPI) and targeted therapies have significantly improved prognosis of patients with various malignancies including those with CNS metastases of melanoma and lung cancer. As part of their multidisciplinary treatment, many patients will be treated with high-dose radiotherapy (RT) to CNS metastases and receive CPI either concurrently or within short time intervals both before or after RT; this combination has been observed to beneficially decrease the incidence of new CNS metastases [83]. On the contrary, CPI have been demonstrated to enhance the risk for symptomatic RN [84]. Most RN occur within the first year after RT [85]

#### Diagnosis ex juvantibus

To the present day, there is no single modality to accurately differentiate between BBD/RN and tumor recurrence, and even when considering the previous points, in some cases, diagnosis can only be made retrospectively. Shortly after beginning treatment with corticosteroids, a decrease in the T2-FLAIR edema can be seen, whereas CE only declines slowly. In patients treated with bevacizumab, a reduction of T2-FLAIR edema as well as CE can be seen quite rapidly. Nevertheless, the central necrotic zone, which represents the damaged brain tissue, is irreversible and remains a gliotic/cystic zone. Treatment with bevacizumab can also reveal or unmark progressive tumor tissue which can be further treated.

## Conclusion

Due to the increasing use of SRS and Re-RT, high-dose treatment at the skull base, and other dose-escalating radiotherapy approaches, detection of new or progressing CEL is encountered more frequently. We therefore propose a detailed nomenclature of treatment-related changes post radiotherapy. We describe the difference between BBD and RN and the pathogenic interplay of both, explaining the possible transition from BBD to RN, if the positive feedback loop is not disrupted early enough. Since there is currently no diagnostic modality to distinguish reliably between brain tissue necrosis, tumor necrosis, and tumor progression, a multistep approach with interdisciplinary consultation to obtain diagnostic accuracy is needed. Because CEL often represents a mixture of tumor cells and radiation injury, the goal may also be to identify the predominant component. Not all CEL require treatment and it is therefore very important to distinguish between posttherapeutic effects (like BBD and RN) and tumor progression.

## Disclaimer

Our recommendations are a resource for professionals involved in the management of RN. The implementation of this guideline requires multidisciplinary structures of care, and defined processes of diagnosis and treatment. These recommendations are a guide and not meant to be prescriptive; ultimately, each physician will need to make treatment decisions based on discussions with the patient. Adherence to this practical guideline will not ensure successful treatment in every situation. This guideline was prepared on the basis of information available at the time the panel was conducting its research and discussions on this topic.
